# Commentary: Experimental Mouse Models of Invasive Candidiasis Caused by *Candida auris* and Other Medically Important *Candida* Species

**DOI:** 10.33696/immunology.4.130

**Published:** 2022

**Authors:** Hong Xin

**Affiliations:** Department of Microbiology, Immunology, and Parasitology, Louisiana State University Health Sciences Center, New Orleans, LA 70112, USA

## Abstract

The study “**“Experimental Mouse Models of Disseminated *Candida auris* Infection”** provides the first insight into the critical role of C5 in the host antimicrobial defense to *disseminated candidiasis caused by C. auris*. This study also establishes an inbred A/J mouse model of systemic *C. auris* infection without drug-induced immunosuppression. *C. auris* has become the first fungal pathogen causing global public health threat due to its multidrug resistance (MDR) and persistence in hospital and nursing home settings. Currently, as compared to *C. albicans*, very limited animal models are available to study the progression of non*-albicans Candida* (NAC) species including *C. auris*. We have successfully established immunosuppressed C57BL/6, BALB/c and A/J murine models of disseminated candidiasis caused by five clinically significant *Candida* species: *C*. *albicans, C. glabrata, C. tropicalis, C. parapsilosis* and *C. auris*. Here we also report updated progress of some important mouse models for *C. auris* infection in the field. These valuable mouse models can be used for the assessment of antifungal drugs, evaluation of potential vaccines and monoclonal antibodies (mAbs) to protect before and after candidiasis, and comparison of pathogenicity of different *Candida* species.

Disseminated candidiasis is the leading cause of life-threatening fungal infections in humans. This is especially the case in immunocompromised individuals, and in hospitalized patients including intensive care unit patients. Despite the availability of modern antifungal therapy, unacceptably high mortality remained same in the last decade [1–4]. The infection is caused by multiple species of the fungal genus *Candida* with *C. albicans* being the most common, together with *C. tropicalis*, *C. glabrata and C. parapsilosis*, causing >95% disseminated candidiasis in humans. Of particular concern, the so called “superbug” ***C. auris*** is a multidrug resistant (MDR), health care-associated super spreading fungal pathogen, and has recently emerged as the first fungal pathogen to cause a global public health threat [5].

A/J mice are naturally deficient in complement protein C5 and its cleaved product C5a, anaphylatoxin and a pro-inflammatory chemoattractant important for anti-*Candida* protection [6–8]. This renders A/J mice highly susceptible to *C. auris* disseminated infection without the need for immunosuppressive drugs [9]. Using this invasive candidiasis model, we recently reported three mAbs that provided significant protection against *C. auris* systemic infection as evidenced by prolonged survival and significantly reduced fungal burdens in the targeted organs as compared to control mice [10]. Overall, our prior studies demonstrate the efficacy of passive transfer with protective mAbs as a novel therapeutics against MDR *C. auris*. Singh et al. also reported that NDV-3A vaccination protected immunosuppressed ICR mice, rendered neutropenic by a combined treatment of cyclophosphamide and cortisone acetate, from lethal *C*. *auris* disseminated infection [11]. To fight *C. auris* invasive infections, there is an urgent demand to test new therapy methods, drugs, and tools. Recently, therapies focused on the synergism of echinocandins with other antifungal drugs was widely explored, representing a novel and promising approach for the treatment of *C. auris* infections [12]. Others reported one of the promising approaches seems to be synergistic interactions of compounds with antifungals. Published data have also focused on the combination therapy as alternative approaches with antifungal drugs open new insights in the management of a global threat of this multidrug-resistant *C. auris*, as described by Srivastava et al. [13].

Although *C. albicans* is still the most commonly isolated Candida species in clinical setting, the prevalence of bloodstream infection with non-*albicans Candida* species increased markedly in recent years due to increasing use of prophylactic antifungal agents. *C. auris*, with its propensity to spread rapidly in critically ill patients and is difficult to treat, has the potential to globally emerge as a dominant opportunistic pathogen in these vulnerable populations. It is believed that the majority of patients with invasive *C. auris* infections have received broad-spectrum antimicrobial agents and, in some cases, antifungal agents prior to the development of invasive candidiasis [14,15]. Unlike *C*. *albicans*, which colonizes the gastrointestinal (GI) and genitourinary tracts of most healthy individuals, *C*. *auris* is hypothesized to predominantly colonize the skin; however, in rare instances, it has been isolated from the gut, oral, and esophageal mucosa of infected individuals [14]. Miyazaki et al. showed the capability of the colonization and dissemination of *C*. *auris* strains by use of an endogenous dissemination mouse model under treatment with cortisone acetate. They also reported important findings that the invasive C. auris strains, especially the South Asian clade, were more effective at colonization and dissemination from the gastrointestinal tract as compared to the non-invasive strains [16]. More interesting and clinically related they demonstrated the biofilm-forming capabilities of the invasive *C. auris* strains were also higher than those of the non-invasive strains, although the detailed factors leading to these differences are unknown. Regardless, even though it is commonly accepted that gastrointestinal colonization of *C*. *auris* is relatively rare compared with skin and nostril colonization due to the poor growth under anaerobic conditions, other groups have reported the similar finding and results of *C*. *auris* gastrointestinal colonization and dissemination under corticosteroid immunosuppression [17,18]. These mouse models are not considered to be directly relevant to human clinical situations, however, the fact that *C*. *auris* could colonize and invade from gastrointestinal tracts under immunosuppressant treatment is regarded as being significant. Clinicians should be aware of the capability of *C*. *auris* colonization and dissemination, especially in immunocompromised patients. Further investigation of innate host immunity in fighting invasive *C. auris* infection and identifying virulence factors necessary for fungal GI colonization and dissemination will be urgently needed. We have established GI colonization and disseminated mouse models for *C. albicans* but not as yet for other NAC [19].

*C. auris* infections can actually occur as community acquired infections as well as infections in the hospital setting where isolates show multi-drug resistance and high mortality [18]. The drug resistance of *C. auris* to the majority of antifungal classes is particularly critical for the therapeutic strategy in high-risk immunocompromised people, for antifungal agents are not effective and generally are not able to clear *Candida* in the immunocompromised people. Given the high mortality rate (40–75%) and significant burden ($4.7 billion/year in U.S.) on the healthcare system associated with disseminated candidiasis [5], novel approaches are needed to supplement or replace current antifungal therapy. During the SARS-CoV-2 pandemic, major outbreaks of invasive *C. auris* infection in hospitalized COVID-19 patients have been reported in the United States. The rapid spread of *C. auris* in hospitalized patients results in high mortality [20–23]; therefore, effective therapeutic strategies must be implemented to avoid the lethal combination of these emerging infectious threats. Recently, cases of *C. auris* candidemia have been reported in pediatric patients with COVID-19 [24]. In comparison to adults, the incidence of invasive candidiasis is even higher in children, with the highest risk in neonates. Miguel’s group recently developed a neonatal mouse model of *C. auris* disseminated infection, in which *C. auris* dissemination was evaluated by fungal burden and histopathological analysis of spleen, kidney liver and brains at different time frames [25]. *Their data showed that among all targeted organs* neonatal liver and brain suffer the worst damage and being the most susceptible tissues to *C. auris* dissemination. This mouse model will be very helpful to deepen the understanding of pathogenesis mechanisms and facilitate strategies for prevention and management of *C. auris* infections in newborns.

Recent epidemiological studies suggest but are not limited to the following risk factors for invasive Candidiasis: Prior administration of broad-spectrum antibiotics and/or antifungal agents, diabetes, surgery (abdominal or vascular), kidney disease, intravenous nutrition, and immunosuppression such as neutropenia, chemotherapy, or transplant related immunosuppression [26–28]. In particular, invasive fungal infections are a major cause of death in organ transplant patients, and the primary pathogens responsible are *Candida* spp., *A. fumigatus* and *Cryptococcus neoformans*. *Especially, Candida* spp. remain the most common invasive fungal infection in solid organ transplant recipients, and mortality is high, ranging from 22 to 44% at 90 days [28]. Emergence of newer species, notably MDR *C. auris*, raises concerns for worsening morbidity and mortality, as it is often resistant to first-line antifungals and capable of efficient nosocomial spread. Recently, a new and clinically relevant transplant immunosuppression model of tacrolimus (FK506) and hydrocortisone-associated pulmonary aspergillosis was developed [29]. A similar mouse model using calcineurin inhibitor as an immunosuppressant is urgently needed for investigating both anti-*Candida auris* immunity and antifungal therapy in organ transplant candidiasis.

Experimental mouse models of candidiasis are critical for understanding fungal pathogenesis and developing host defense strategies against the infection. Such models enable the evaluation of potential antifungal vaccines and assessing efficacy of chemotherapies. The fungus *Candida albicans* and other related *Candida* species are commensal organisms in healthy humans but become opportunistic pathogens in immunocompromised patients. To investigate systemic *Candid*a infection, mouse models have been developed with the aim of mimicking the clinical setting of human diseases and the most common being the intravenous infection model. The murine intravenous model of disseminated *C. albicans* infection has been well studied and characterized, while reproducible and reliable mouse models for *NAC* are lacking because most *NAC* species are generally nonpathogenic in mice. Therefore, developing faithful mouse models to understand host immunity to NAC species is critical for disease control and prevention. We have successfully established murine models of intravenous disseminated infection by *C. tropicalis*, *C. glabrata, C. parapsilosis* and *C. auris,* in addition to *C. albicans.* With the aim of mimicking the clinical situations in humans as closely as possible, experimental *Candida* infections were usually induced in immunocompromised mice by pretreatment with cytotoxic agents, such as cyclophosphamide (CY) [28–31] or cortisone acetate treatments [30–33]. We have developed and established different intravenous models of disseminated infection by all above medically important *NAC* species in immunosuppressed C57BL/6, BALB/c and A/J strains (Table 1). Briefly, for disseminated *C. tropicalis* and *C. parapsilosis* infection, a combination of a large inoculum and immunosuppression [200 mg/kg dose of cyclophosphamide (CY) by intraperitoneal (i.p.) administration given weekly] was enough to establish severe acute infection in C57BL/6 and BALB/c mice. However, this strategy was not a prerequisite for *C. glabrata* disseminated candidiasis, for CY-treated mice were still resistant against systemic infection by *C. glabrata* and even survived with high fungal burdens in the kidney. We then further immunosuppressed mice via a combination of CY treatment with cortisone acetate given daily subcutaneously (s.c.) followed by high inoculum of *C. glabrata* (1×10^8^ challenge, and eventually achieved 70% mortality within 20 days post-infection in mice [33]. We also established an A/J mouse model of systemic *C. auris* infection without immunosuppression [9], and a C57BL/6 model with immunosuppressant (Table 1). The established immunocompromised mouse models closely mimic the immunocompromised patient situations and are valuable tools for evaluating *in vivo* efficacy of mAb and antifungal immunotherapies.

These murine models of intravenous disseminated infection by NAC listed above used a combination of cyclophosphamide and cortisone acetate, suppressing both innate and adaptive immunity. Terres et al. recently reported an alternative approach, and compared *C. auris* infection in two neutrophil-depleted BALB/c models in which innate immunity is targeted using mAb 1A8 and RB6–8C5. 1A8 is an anti-Ly6G antibody that depletes neutrophils, and RB6–8C5 is an anti-Ly6G/Ly6C antibody that depletes Ly6G+ and Ly6C+ neutrophils, dendritic cells, and subpopulations of lymphocytes and monocytes. The different inoculums of 10^7^ and 10^8^ as well as the intravenous and gavage routes of infection were also investigated. The results reveal that neutrophil depletion in BALB/c mice is sustained long-term with the 1A8 antibody and short-term with RB6–8C5, however, the kidney, heart, and brain were the organs with the greatest fungal burdens, regardless of which neutrophil-depleting antibody was used [34].

Other reported discrepancies in the pathogenesis and progress of *C. auris* in host reported by different research groups are mostly due to differences in the genetic background of mouse strains used, variances in the route of dissemination, and the various treatments of immunosuppressant. As susceptibility to systemic *C*. *auris* infection varies among mouse strains, we must be aware that the strains used in our and others’ studies (C57BL/6, BALB/c, ICR and C57BL/6J) have shown to be much more resistant to *C*. *auris* infection than A/J mice, a C5-deficient inbred strain. In addition, genetic mouse models of human immunodeficiencies can be good candidates for investigating the protective mechanism of immunotherapy to control disseminated candidiasis. The association of C5 with greater susceptibility in A/J mice to NAC invasive infection provides strong evidence that C5 may play a critical role for enhanced host resistance and innate immunity to *Candida* systemic infections. Therefore, to establish a reliable and simple mouse model of acute disseminated candidiasis, the C5 status of the mouse strains should be considered as an important predictor of their resistance to the disease. Invasive *C*. *auris* infection is associated with high mortality rates, and often resistant to multiple classes of antifungals. The biggest challenge we are facing is inefficient antifungal drugs in immunocompromised patients. Clinical experience tells us that novel approaches are needed to supplement or replace current antifungal therapy. Indeed, a better understanding of the host–fungi interaction is invaluable for the development of new prophylactics or therapies. Animal models provide a useful tool to study the response to systemic candidiasis and evaluate antifungal therapy. Studies in mice have indicated the importance of the innate immune system, particularly the complement pathway, in controlling invasive fungal infection [9,34,35]. Meanwhile, long-lasting resistance to fungal infection due to acquired immunity is also very important, and is induced by specific antigens initiating inflammatory immune response, humoral responses, and phagocytic cell activation. However, important differences between mice and humans must be taken into account when interpreting experimental data. For example, mice and humans differ with respect to their commensal fungal gut flora. Furthermore, known discrepancies exist in both innate and adaptive immunity between human and mice, including: balance of leukocyte subsets, Toll-like receptors, cytokine receptors, Th1/Th2 differentiation, and much more [36]. Such differences should be taken into account, as the conclusions drawn from mouse studies depend on a critical understanding of the inherent limitations and parallels of mice as preclinical models for human disease.

## Figures and Tables

**Table 1:** Intravenous C57BL/6, BALB/c and A/J mouse models of disseminated candidiasis.

*Candida strain*	Challenge dose^1^	Mouse strain
***C. albicans* SC5314 (ATCC MYA-2876)**	2×10^6^/5×10^5^	C57BL/6 / BALB/c
***C. tropicalis* (ATCC200956)** ^2^	1×10^8^	C57BL/6; A/J; BALB/c (CY i.p. treatment)
***C. glabrata* (ATCC 200918)** ^3^	1 ×10^8^	C57BL/6 & BALB/c (CY^2^ by i.p. weekly + cortisone by s.c. daily)
***C. glabrata* (ATCC 200918)**	1×10^8^	A/J (CY i.p. treatment)
***C. auris* (AR-CDC 0386)**	2×10^8^	A/J; C57BL/6 & BALB/c (CY i.p. treatment),
***C. parapsilosis* ATCC22019**	1×10^7^	C57BL/6 & BALB/c (CY i.p. treatment)

1 The optimal doses of each *Candida* strain for producing an acute infection with 60–100% of animals dying within 10–20 days in C57BL/6, BALB/c and A/J mouse strains

2 *C. tropicalis* (ATCC200956); *amphotericin B-resistant*

3 *C. glabrata* (ATCC 200918); fluconazole-resistant
